# Long-term outcome of intravitreal anti-vascular endothelial growth factor treatment for pachychoroid neovasculopathy

**DOI:** 10.1038/s41598-021-91589-2

**Published:** 2021-06-08

**Authors:** Jihyun Yoon, Wontae Yoon, Seung kwan Na, Jihyun Lee, Chul Gu Kim, Jong Woo Kim, Han Joo Cho

**Affiliations:** Kim’s Eye Hospital, Konyang University College of Medicine, 156, 4ga, Yeoungdeungpo-dong, Yeoungdeungpo-gu, Seoul, South Korea

**Keywords:** Retinal diseases, Outcomes research, Macular degeneration

## Abstract

To compare the long-term effectiveness of intravitreal anti-vascular endothelial growth factor (VEGF) treatment for pachychoroid neovasculopathy (PNV), polypoidal choroidal vasculopathy/aneurysmal type 1 neovascularization (PCV/AT1), and typical neovascular age-related macular degeneration (nAMD). Forty-one eyes with PNV, 68 eyes with PCV/AT1, and 56 eyes with typical nAMD were retrospectively included for analysis. All patients were treatment-naïve and received a three-monthly loading injection of anti-VEGF, followed by further injections, as required. The visual and anatomical outcomes after treatment were evaluated up to 36 months from baseline. No significant intergroup difference was found in terms of best-corrected visual acuity (BCVA) and changes in central foveal thickness at 12, 24, and 36 months after the baseline. In addition, no significant difference was found between the groups regarding the proportions of improved or worsened (increased or decreased more than 3-lines) visual acuity. However, the PNV group participants received significantly fewer anti-VEGF injections (11.7 ± 6.9) than those in the PCV/AT1 (12.4 ± 7.0; *P* = 0.031) and typical nAMD groups (13.2 ± 7.4; *P* = 0.016). The incidence of macular atrophy (MA) development was also significantly lower for the PNV (4/41 eyes, 9.8%) than the typical nAMD (15/56 eyes, 26.8%; *P* = 0.033) eyes. There was no significant difference between PNV, PCV/AT1, and typical nAMD regarding visual acuity improvement after anti-VEGF treatment over 36 months. However, the number of injections for PNV was significantly lower compared to that for PCV/AT1 and typical nAMD, and the incidence of MA development was significantly lower than in typical nAMD.

## Introduction

Age-related macular degeneration (AMD) is a leading cause of irreversible visual loss in elderly people^1^. AMD associated with choroidal neovascularization (CNV), which causes subretinal exudative changes and hemorrhage, is called neovascular AMD (nAMD).

Recently, it has been suggested that pachychoroid neovasculopathy (PNV) is a type 1 (sub-retinal pigment epithelium [RPE]) CNV characterized by underlying dilated choroidal vessels, attenuation of choriocapillaris, and thickened choroid^2^. PNV has been proposed as one of the pachychoroid-driven spectrum of diseases, including pachychoroid pigment epitheliopathy (PPE)^3^, central serous chorioretinopathy (CSC), and polypoidal choroidal vasculopathy/aneurysmal type 1 macular neovascularization (PCV/AT1)^2,4,5^.

Phenotypically, PNV is distinguished from typical nAMD by the relatively younger age, paucity of drusen, and diffuse increase in choroidal thickness and dilation of the outer choroidal vessels compared to typical nAMD^2,6^. Recent evidence suggests the possibility of different genetic backgrounds between PNV and typical nAMD. An investigation including 200 Japanese patients found that the genetic susceptibility (*ARMS2* and *CFH*) of PNV was significantly lower than that of typical nAMD^7^. Another study of single nucleotide polymorphisms in 201 Caucasians with nAMD suggested that the risk alleles that predispose patients to neovascularization in the pachychoroid cohort are not necessarily specific to the context of AMD^8^. Differences in the pathophysiology of CNV among typical nAMD, PNV, and PCV/AT1 have been noted in many published reports; however, such differences are still controversial.

Currently, PNV is treated with anti-vascular endothelial growth factor (VEGF) in the same way as nAMD without distinction because PNV harbors CNV^2,7,9^. Several investigations have reported the favorable outcome of anti-VEGF treatment for PNV^10–12^. However, to date, long-term evaluation of the treatment efficacy of anti-VEGF for PNV has not been sufficiently reported. The purpose of the current study was to evaluate the long-term therapeutic efficacy of anti-VEGF treatment for PNV and to compare the therapeutic results with those of other types of nAMD.

## Methods

The patients diagnosed with nAMD and treated with anti-VEGF (ranibizumab or aflibercept) between January 2016 and August 2017 were identified by computerized searching of the electronic medical charts. All patients were diagnosed and treated at the Retina Center of Kim’s Eye Hospital, Konyang University College of Medicine. The current research followed the tenets of the Declaration of Helsinki, and was approved by the institutional review board at Kim’s Eye Hospital. The need for informed consent was waived by the Institutional Review Board.

### Subjects

The inclusion criteria were as follows: (1) over 50 years old; (2) confirmation of n AMD with spectral-domain optical coherence tomography (SD-OCT, Spectralis®; Heidelberg Engineering, Dossenheim, Germany), fluorescein angiography (FA), and indocyanine angiography (ICGA; HRA-2, Heidelberg Engineering) at the first visit; (3) no previous anti-VEGF treatment; and (5) a minimum follow-up period of 36 months. When a case of bilateral nAMD was encountered, only the eye earlier diagnosed was included.

**Figure 1:**
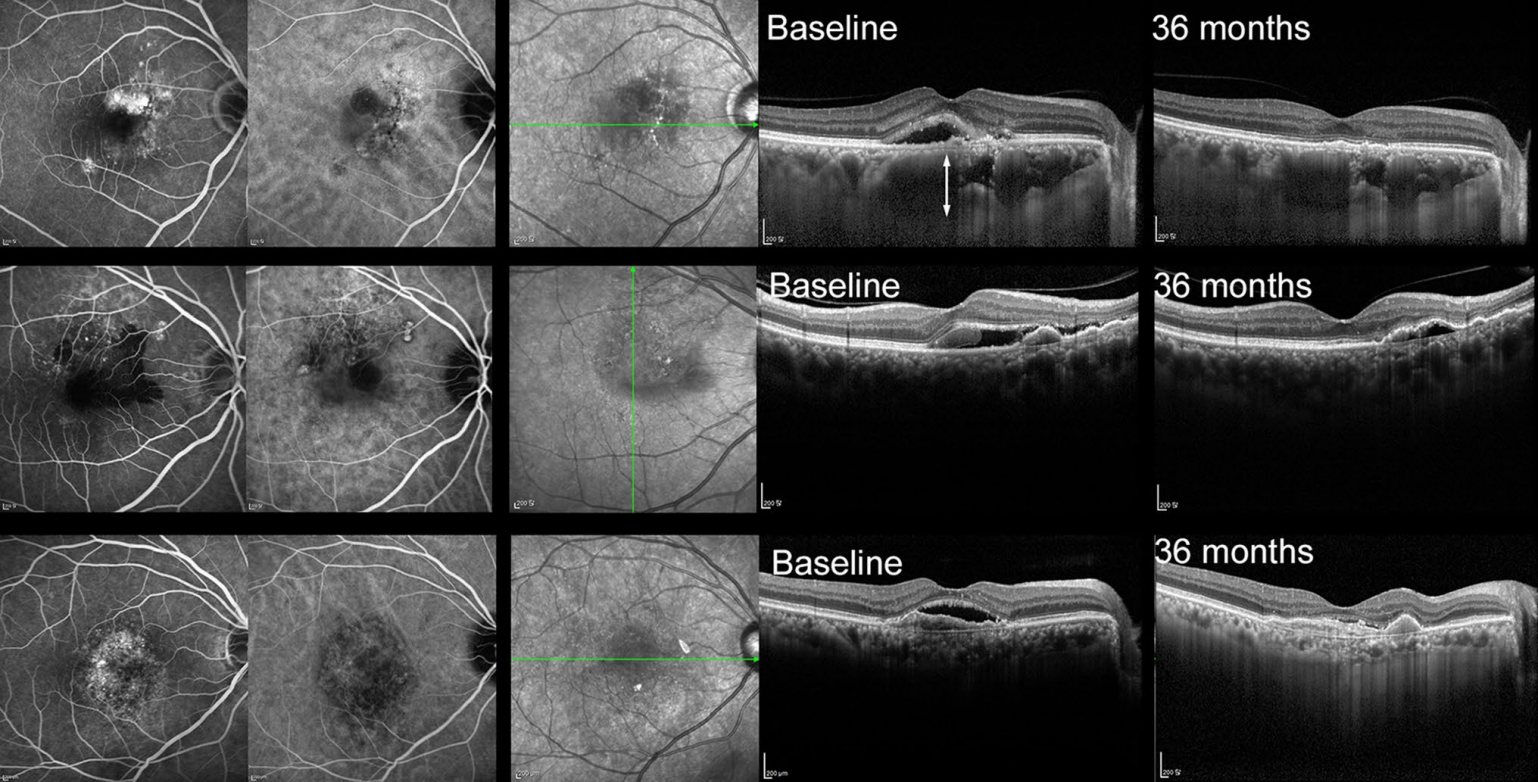
Representative cases of pachychoroid neovasculopathy (PNV), polypoidal choroidal vasculopathy/aneurysmal type 1 neovascularization (PCV/AT1), and typical neovascular age-related macular degeneration (nAMD) treated with anti-vascular endothelial growth factor. (upper line) A 56-year-old male patient with PNV treated with only 7 injections of aflibercept with 36 months of follow-up. A dilated choroidal vessel is noted under the macular neovascularization (upper line, second image, between the white arrowhead). (middle line) A 66-year-old male patient with PCV/AT1 treated with 12 injections of aflibercept injection over 36 months. (bottom line) A 70-year-old male patient with typical nAMD treated with 15 injections of aflibercept over 36 months.

All the eyes were classified as PNV, PCV/AT1, and typical nAMD after reviewing the medical records of the patients with nAMD. The definition of PNV was based on the latest reports^2,10,13,14^, since it is still evolving. PNV was diagnosed using the following criteria: (1) definite type 1 CNV on OCT and ancillary images, including FA/ICGA images; (2) no drusen in both eyes (no AMD, AREDS category; (3) presence of dilated choroidal vessels below the macular neovascularization; and (4) PPE or CSC characteristics including choroidal vascular hyperpermeability (CVH) on ICGA^15^, or a history of CSC. When it was difficult to ascertain whether a shallow irregular RPE elevation was a true type 1 macular neovascularization or not, OCT angiography was used for confirmation. The diagnosis of PCV/AT1 was based on the definite presence of polypoidal lesions with or without branching vascular networks in the ICGA images. When the polypoidal lesion could not be evaluated due to severe subretinal hemorrhage at baseline, the lesion was re-evaluated after the loading injection, following repeated ICGA. The representative cases are shown in Fig. 1.

The exclusion criteria were as follows: (1) type 3 macular neovascularization (also known as retinal angiomatous proliferation [RAP]); (2) end-stage nAMD with macular fibrosis or geographic atrophy (GA) at baseline; (3) treated with concomitant photodynamic therapy; and (4) presence of other concomitant ocular diseases, such as diabetic retinopathy or retinal vein occlusion.

As a routine for nAMD treatment in our institution, all the patients were administered three loading injections of anti-VEGF (ranibizumab [0.5 mg/0.05 mL] or aflibercept [2 mg/0.05 mL]) at monthly intervals. After the initial loading injections, the patients were followed up during the study period at 4 to 8-week intervals, and they received further injections as required. All the patients underwent standardized examinations at every visit, including best-corrected VA (BCVA), fundus examination, SD-OCT (consisting of 19 or 31 horizontal lines [6 mm × 6 mm area]), and additional FA/ICGA, OCT angiography, or autofluorescence (AF) at the discretion of the physician.

### Patient assessment and outcome measures

The main visual outcome was the mean change in best-corrected visual acuity (BCVA; logarithm of the minimal angle of resolution [logMAR] converted from Snellen BCVA) from baseline to 3, 12, 24, and 36 months. The proportion of patients who gained or lost more than three lines of BCVA compared to the baseline was also recorded.

The main anatomical outcome was the mean change in central foveal thickness from baseline to 3, 12, 24, and 36 months. Central foveal thickness was measured between the vertical distance from the hyperreflective line of Bruch’s membrane to the inner limiting membrane on the fovea-centered SD-OCT images. In addition, the development of macular atrophy (MA) in the patients was evaluated during the 36-month follow-up period. Presence of MA was detected via color fundus photography (CFP), SD-OCT, infrared reflectance image, and autofluorescence images (AF). MA was defined as in our previous investigations^16,17^: (1) well-demarcated, hypopigmented area with a minimum linear dimension of 250 µm; (2) occurred within the macular vascular arcades; (3) uniformly reduced autofluorescence signals on AF or increased visibility of the underlying choroidal vessels; and (4) confirmation of increased signal transmission on the SD-OCT findings due to the absence of RPE.

All measurements were estimated using the Heidelberg Eye Explorer software (v. 5.6.4.0; Heidelberg Engineering) by two retinal specialists (J.L. and S.K.N.) who were blinded to the patient information. When the evaluation was inconsistent for the diagnosis of PNV between the graders, a senior investigator (H.J.C.) made the final decision after an open discussion.

### Statistical analysis

SPSS for Windows, Version 18.0 (SPSS Inc., Chicago, IL, USA) was used for all the statistical analyses. Frequencies were compared between the groups by using the chi-square or Fischer’s exact test. One-way analysis of variance (ANOVA) was used to compare continuous variables between the groups. The Bonferroni correction was used to make statistical adjustments for multiple comparisons. Inter-grader agreement was measured using Cohen’s Kappa coefficient. A P-value of less than 0.05 was considered statistically significant.

## Results

### Baseline characteristics

In total, 165 eyes (165 patients) met the inclusion criteria and were enrolled in the study for analysis. After reviewing the patients’ medical records, 41 eyes were categorized into the PNV group, 68 eyes into the PCV/AT1 group, and 56 eyes into the typical nAMD group. The intra-grader agreement between the graders for the diagnosis of PNV showed an almost perfect agreement (the Cohen’s Kappa was 0.88). All patients were South Koreans, and the clinical details of the enrolled patients are presented in Table 1.

**Table 1:** Baseline characteristics of patients treated with anti-VEGF for macular neovascularization.

	PNV (41 eyes)	PCV/AT1 (68 eyes)	Typical nAMD (56 eyes)	*P*
**Age (years) (mean ± SD)**	66.30 ± 8.12	70.77 ± 7.12	73.55 ± 7.31	0.002^a^
**Sex**				0.649^b^
Male	25 (61.0%)	40 (58.8%)	31 (55.4%)	
Female	16 (39.0%)	28 (41.2%)	25 (44.6%)	
**Baseline BCVA (logMAR)** (Snellen equivalent)	0.39 ± 0.34 (20/49)	0.42 ± 0.31 (20/52)	0.46 ± 0.33 (20/57)	0.231^a^
< 0.40 (20/40)	9 (22.0%)	14 (20.6%)	12 (21.4%)	0.898 ^b^
0.40 (20/40) to 1.0 (20/200)	24 (58.5%)	35 (51.5%)	29 (51.8%)	
> 1.0 (20/200)	8 (19.5%)	19 (27.9%)	15 (26.8%)	
**Mean baseline central foveal thickness ± SD (µm)**	362 ± 188	379 ± 102	397 ± 184	0.612^a^
**Mean subfoveal choroidal thickness ± SD (µm)**	349 ± 103	337 ± 109	272 ± 126	< 0.001^a^
**Lesion location, n (%)**				0.503^b^
Foveal (subfoveal and juxtafoveal)	28 (68.8%)	49 (72.1%)	44 (78.6%)	
Extrafoveal	13 (31.2%)	19 (27.9%)	12 (21.4%)	
**Mean lesion size ± SD(mm**^**2﻿**^**﻿)**	2.26 ± 1.87	2.31 ± 1.66	2.51 ± 1.54	0.319^a^
**Choroidal vascular hyperpermeability, n (%)**	25 (61.0%)	41 (60.3%)	12 (21.4%)	< 0.001^b^
**Feature of fluid at baseline**				
SRF	35 (85.4%)	60 (88.2%)	42 (75.0%)	0.133^b^
IRF	5 (12.2%)	26 (38.2%)	27 (48.2%)	0.001^b^
Retinal hemorrhage	5 (12.2%)	35 (51.4%)	17 (30.4%)	< 0.001^b^
**Anti-VEGF agent**				
Ranibizumab	11 (26.8%)	14 (20.6%)	15 (26.8%)	0.782^b^
Aflibercept	25 (61.0%)	44 (64.7%)	35 (62.5%)	
Both^c^	5 (12.2%)	10 (14.7%)	6 (10.7%)	

^a^Based on one-way analysis of variance.

^b^Based on chi-square test.

^c^Patients for whom the anti-VEGF treatment was switched during the study period; there was a switch from ranibizumab to aflibercept for 14 eyes (66.7%) and a switch from aflibercept to ranibizumab for the others.

*BCVA* best-corrected visual acuity, *IRF* intraretinal fluid, *logMAR* logarithm of the minimum angle of resolution, *nAMD* neovascular age-related macular degeneration, *PCV/AT1* polypoidal choroidal vasculopathy/aneurysmal type 1 neovascularization, *PNV* pachychoroid neovasculopathy, *SD* standard deviation, *SRF* subretinal fluid, *VEGF* vascular endothelial growth factor.

No significant intergroup difference was observed regarding sex, baseline BCVA, baseline central foveal thickness, lesion location, and lesion size (Table 1). However, significant differences between the groups were identified with respect to age, subfoveal choroidal thickness, presence of choroidal vascular hyperpermeability, and features of fluids at baseline (Table 1).

The PNV group was significantly younger (66.30 ± 8.12) than the PCV/AT1 (70.77 ± 7.12) and typical nAMD (73.55 ± 7.31) groups (ANOVA, *P* = 0.002*,* Table 1). The average subfoveal choroidal thickness of the PNV (349 ± 103 µm) group was thicker than that of the typical nAMD group (272 ± 126 µm, Bonferroni correction for multiple comparison, *P* = 0.012). The average subfoveal choroidal thickness between PNV and PCV/AT1 (337 ± 109 µm) showed no significant difference (Bonferroni correction for multiple comparison, *P* = 0.510). Choroidal vascular hyperpermeability was significantly frequent for PNV (25/41eyes, 61.0%) and PCV/AT1 (41/68 eyes, 60.3%) than for typical nAMD (12/56 eyes, 21.4%) (*P* < 0.001, Table 1).

The feature of exudative fluid at baseline was significantly different between the groups. No significant difference was found regarding the incidence of subretinal fluid (SRF) between the groups, However, the PNV group showed a significantly lower incidence of intraretinal fluid (IRF, 5/41 eyes, 12.2%) and retinal hemorrhage (5/41 eyes, 12.2%) at baseline compared to the PCV/AT1 (26/68 eyes, 38.2% for IRF; 35/68 eyes, 51.4% for retinal hemorrhage) and typical nAMD (27/56 eyes, 48.2% for IRF; 17/56 eyes, 30.4% for retinal hemorrhage) groups (*P* = 0.001 for IRF and *P* < 0.001 for retinal hemorrhage, respectively, Table 1).

### Visual and anatomical outcomes

**Figure 2:**
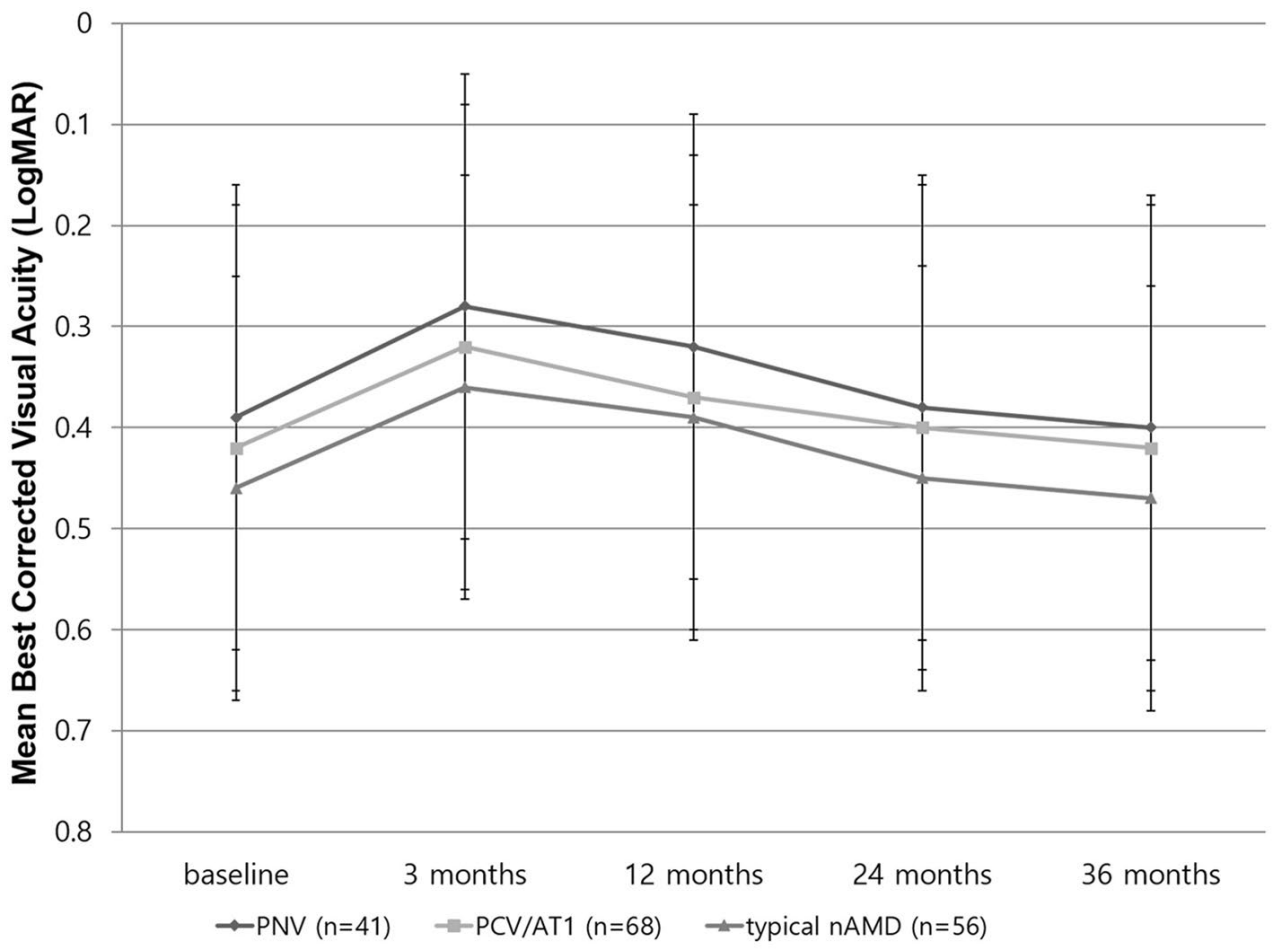
Changes in mean best-corrected visual acuity (BCVA), expressed as logarithm of the minimal angle of resolution, during the 36-month anti-vascular endothelial growth factor treatment for macular neovascularization. The BCVA at 3 months was significantly different between the pachychoroid neovasculopathy (PNV), polypoidal choroidal vasculopathy/aneurysmal type 1 neovascularization (PCV/AT1), and typical neovascular age-related macular degeneration (nAMD) (ANOVA, P = 0.031). However, there was no significant difference between the groups at 12, 24, and 36 months.

The mean BCVA change from the baseline to 36 months was similar between the PNV, PCV/AT1, and typical nAMD groups during the anti-VEGF treatment (Fig. 2). The BCVA of PNV improved from 0.39 ± 0.34 (Snellen equivalent; 20/49) at baseline to 0.30 ± 0.23 (20/39, *P* = 0.013) at 3 months; 0.33 ± 0.27 (20/42, *P* = 0.039) at 12 months; 0.39 ± 0.26 (20/49, *P* = 0.821) at 24 months; and 0.40 ± 0.27 (20/41, *P* = 0.554) at 36 months. The BCVA of PCV/AT1 improved from 0.42 ± 0.31 (20/52) at baseline to 0.32 ± 0.25 (20/41, *P* = 0.015) at 3 months; 0.37 ± 0.29 (20/46, *P* = 0.041) at 12 months; 0.40 ± 0.31 (20/50, *P* = 0.821) at 24 months; and 0.42 ± 0.33 (20/52, *P* = 0.354) at 36 months. The BCVA of typical nAMD improved from 0.46 ± 0.33 (20/57) at baseline to 0.36 ± 0.27 (20/45, *P* = 0.021) at 3 months; 0.39 ± 0.28 (20/49, *P* = 0.040) at 12 months; 0.45 ± 0.36 (20/56, *P* = 0.771) at 24 months; and 0.47 ± 0.37 (20/59, *P* = 0.456) at 36 months. For all groups, a significant improvement of BCVA was found at 3 and 12 months; however, the BCVA at 24 and 36 months showed no significant difference compared to those of the baseline.

The BCVA at 3 months was significantly different between the groups (ANOVA, *P* = 0.026); Bonferroni correction showed that the PNV group showed better BCVA compared to typical nAMD (*P* = 0.040), and no difference compared to PCV/AT1 (*P* = 0.220). However, no difference was found between PCV/AT1 and typical nAMD (P = 0.057). Moreover, no significant difference of BCVA between groups was found at 12 months (ANOVA, *P* = 0.327), 24 months (*P* = 0.118), and 36 months (*P* = 0.059).

After the anti-VEGF treatment for 36 months, there was no difference between the groups in terms of the proportion of eyes that achieved a BCVA of 20/40 or better at 36 months (PNV; 16/41 eyes, 39.0%, PCV/AT1; 21/68 eyes, 30.9%, typical nAMD; 17/56 eyes, 30.4%; *P* = 0.611) or the proportion of eyes with BCVA of 20/200 or worse at 36 months (PNV; 7/41 eyes, 17.1%, PCV/AT1; 13/68 eyes, 19.1%, typical nAMD; 15/56 eyes, 26.8%; *P* = 0.864, Table 2). Similarly, the proportion of eyes that improved by more than three lines of BCVA was not significantly different between the groups (PNV; 15/41 eyes, 36.6%, PCV/AT1; 23/68 eyes, 33.8%, typical nAMD; 15/56 eyes, 26.8%, Table 2; *P* = 0.550). The proportion of eyes that worsened by more than three lines of BCVA was also not significantly different between the groups (PNV; 10/41 eyes, 24.4%, PCV/AT1; 21/68 eyes, 30.9%, typical nAMD; 14/56 eyes, 25.0%, Table 2; *P* = 0.682).

**Table 2:** Three-year results of the treatment with anti-VEGF for macular neovascularization.

	PNV (41 eyes)	PCV/AT1 (68 eyes)	Typical nAMD (56 eyes)	*P*
**Mean BCVA at 36 months (logMAR [Snellen equivalent])**	0.40 ± 0.27 (20/50)	0.42 ± 0.33 (20/52)	0.47 ± 0.37 (20/59)	0.079^a^
**Mean central foveal thickness at 36 months (µm)**	196 ± 109	218 ± 151	231 ± 147	0.335^a^
**BCVA ≥ 20/40, n (%)**	16 (39.0%)	21 (30.9%)	17 (30.4%)	0.611^b^
**BCVA ≤ 20/200, n (%)**	7 (17.1%)	13 (19.1%)	12 (21.4%)	0.864^b^
**BCVA changes, n (%)**				
Improved ≥ 3 lines (logMAR 0.3)	15 (36.6%)	23 (33.8%)	15 (26.8%)	0.550^b^
Stable	16 (39.0%)	24 (35.3%)	27 (48.2%)	
Worsened ≥ 3 lines (logMAR 0.3)	10 (24.4%)	21 (30.9%)	14 (25.0%)	0.682^b^
**Development of macular atrophy, n (%)**	4 (9.8%)	7 (10.3%)	15 (26.8%)	0.033^b^
**Number of anti-VEGF injections ± SD**	11.7 ± 6.9	12.4 ± 7.0	13.2 ± 7.4	0.023^a^

^a^ Based on one-way analysis of variance.

^b^ Based on chi-square test.

*BCVA* best-corrected visual acuity, *logMAR* logarithm of the minimum angle of resolution, *nAMD* neovascular age-related macular degeneration, *PCV/AT1* polypoidal choroidal vasculopathy/aneurysmal type 1 neovascularization, *PNV* pachychoroid neovasculopathy, *SD* standard deviation, *VEGF* vascular endothelial growth factor.

**Figure 3:**
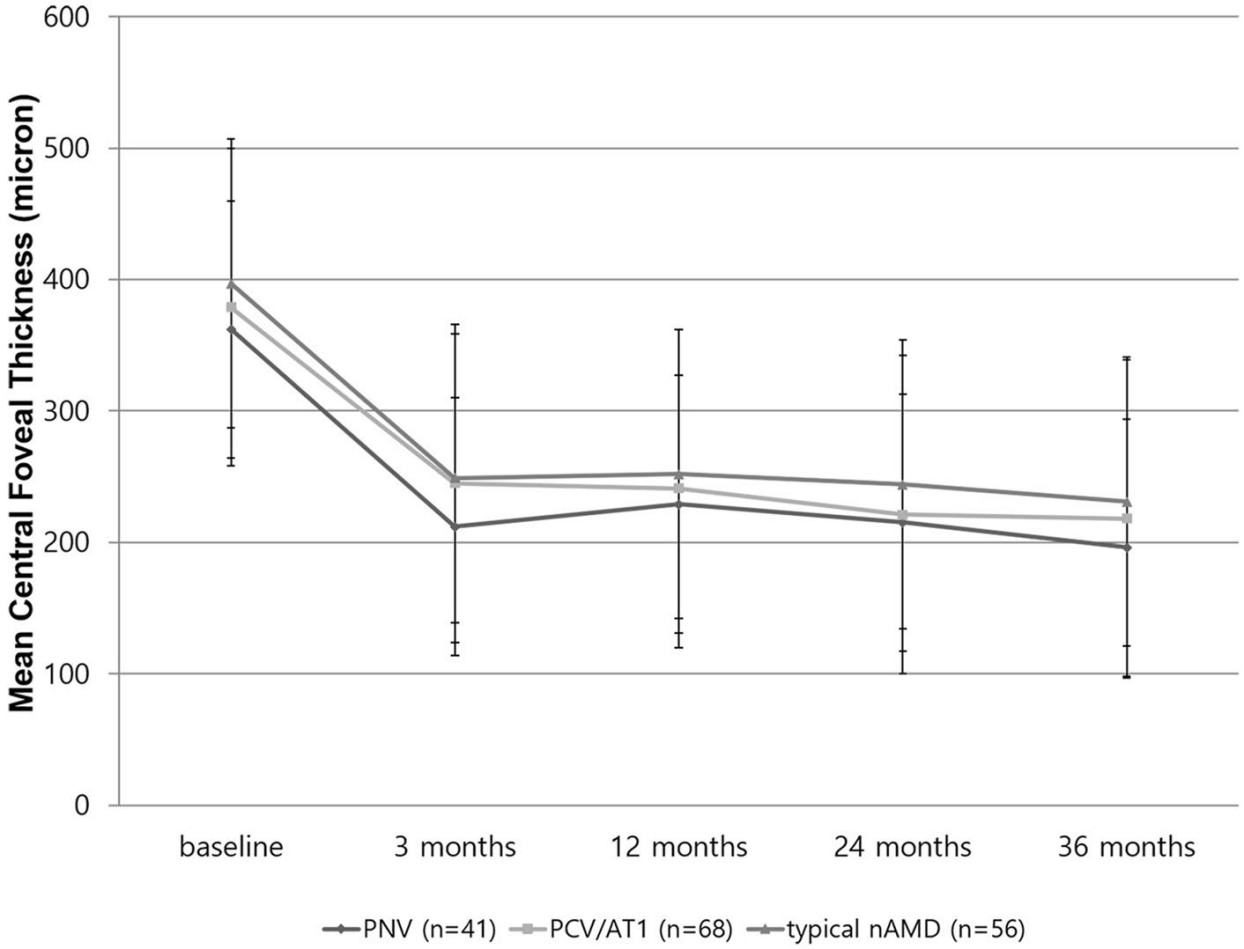
Changes in mean central foveal thickness during the 36-month anti-vascular endothelial growth factor treatment for pachychoroid neovasculopathy (PNV), polypoidal choroidal vasculopathy/aneurysmal type 1 neovascularization (PCV/AT1), and typical neovascular age-related macular degeneration (nAMD). The mean central foveal thickness showed a significant decrease when comparing from baseline through the 36 months follow-up for the groups. However, there was no significant difference between the groups in central foveal thickness at 3, 12, 24, and 36 months.

The mean central foveal thickness of the three groups showed comparable changes between the groups (Fig. 3). The mean central foveal thickness of the PNV group significantly improved from 362 ± 188 µm at baseline to 212 ± 113 µm (*P* < 0.001) at 3 months; 229 ± 133 µm (*P* = 0.002) at 12 months; 215 ± 128 µm (*P* = 0.003) at 24 months; and 196 ± 109 µm (*P* = 0.015) at 36 months. The mean central foveal thickness of the PCV/AT1 group significantly improved from 337 ± 109 µm at baseline to 245 ± 123 µm (*P* < 0.001) at 3 months; 241 ± 153 µm (*P* = 0.004) at 12 months; 221 ± 148 µm (*P* = 0.008) at 24 months; and 218 ± 151 µm (*P* = 0.011) at 36 months. The mean central foveal thickness of the typical nAMD group also significantly improved from 397 ± 184 µm at baseline to 249 ± 153 µm (*P* < 0.001) at 3 months; 252 ± 183 µm (*P* = 0.009) at 12 months; 244 ± 198 µm (*P* = 0.010) at 24 months; and 231 ± 147 µm (*P* = 0.0016) at 36 months. The mean central foveal thickness showed significant decrease through the 36-month follow-up for all groups. However, no significant intergroup difference was found at 3 months (ANOVA, *P* = 0.659), 12 months (*P* = 0.344), 24 months (*P* = 0.221), and 36 months (*P* = 0.311).

The injection number during the 36 months showed a significant difference between the groups (ANOVA, *P* = 0.023, Table 2). Bonferroni correction showed that the injection number of PNV (11.7 ± 6.9) was significantly lower than the typical nAMD (13.2 ± 7.4, *P* = 0.016, and PCV/AT1 (12.4 ± 7.0, *P* = 0.031). No significant difference between the PCV/AT1 and typical nAMD groups was found (*P* = 0.057).

The incidence of MA development during the study period was different between the groups. The incidence was significantly lower for the PNV (4/41 eyes, 9.8%) and PCV/AT1 (7/68 eyes, 10.3%) than the typical nAMD (15/56 eyes, 26.8%; *P* = 0.033, Table 2) groups.

## Discussion

In the current study, anti-VEGF treatment for PNV showed similar long-term visual and anatomical outcomes compared to PCV/AT1 and typical nAMD. Although the BCVA of the PNV group at three months tended to be better than that of the PCV/AT1 and typical nAMD groups, with statistical difference, thereafter, the BCVA at 12, 24, and 36 months showed no difference between the groups. No significant difference was found regarding the proportion of improved and worsened (increased or decreased more than 3-lines) visual acuity, at 36 months, between the groups. Notably, the visual outcome was achieved in the PNV group with fewer injections compared to the PCV/AT1 and typical nAMD groups.

Recently, our study group reported a low recurrence rate of PNV with fewer injections during 12 months of follow-up^10^. Another investigation has reported that the PNV group received fewer aflibercept injections than the type 1 nAMD group with the treat-and-extend (TNE) regimen^18^. The current study also showed that the PNV group received fewer anti-VEGF injections during the 36 months of long-term follow-up. Our results suggest that the response to anti-VEGF treatment may differ in PNV and other types of nAMD.

Recent evidence suggests that there are distinct profiles of VEGF and cytokines between PNV and other types of nAMD. Hata et al. reported that the mean VEGF concentration of PNV was significantly lower than that of nAMD^19^. Kato et al. reported elevated VEGF levels without elevated complement component 3a (C3a) and macrophage chemoattractant protein 1 (MCP-1) for PNV, compared to typical nAMD^14^. Furthermore, in PNV patients, a lack of correlation between cytokine levels and anti-VEGF treatment response has been noted, whereas a negative association is typical in nAMD patients^13^. These differences in VEGF and cytokine profiles between PNV and typical nAMD could have led to the different therapeutic efficacies of anti-VEGF, or the mechanism by which VEGF is involved in angiogenesis.

The feature of baseline fluid also might affect the results. IRF was significantly less frequent for PNV than for PCV/AT1 and typical nAMD in this study. Given that IRF is associated with lower baseline visual acuity^20^, delayed response to anti-VEGF treatment^21^, more frequent injections than SRF^22^, and poor visual outcome^23^, the significant infrequency of IRF in the PNV group could have affected the injection frequency.

Interestingly, although it has been proposed that PNV and PCV/AT1 share clinical phenotypes in many aspects except the presence of polypoidal lesions^24^, the injection number in PNV was also lower compared to PCV/AT1. Presence of polypoidal lesions is a hallmark of PCV/AT1, and a major cause of retinal hemorrhage, exudation, and pigment epithelial detachment (PED)^25^. Often, retinal hemorrhage from the rupture of the polypoidal lesion might need an intensive anti-VEGF treatment^25^. Thus, the paucity of retinal hemorrhage in the PNV than the PCV/AT1 groups could be associated with the fewer injections in the PNV group. Further investigation is needed regarding the reason for the different frequency of anti-VEGF injection, including the difference in pathophysiology between PNV and other types of nAMD.

The incidence of MA was significantly lower in the PNV group than in the typical nAMD group, being similar to that of PCV/AT1 in this study. The development of MA during anti-VEGF treatment for nAMD is an unwanted side effect of the therapy. Once RPE atrophy has developed, it could influence the visual outcome over a long-term follow-up period because the lesion tends to increase in size over time^16^. Furthermore, the risk of developing MA raises the possibility that the visual benefit achieved from anti-VEGF treatment may not be maintained in the long term. It is well known that the incidence of MA is different for the subtypes of nAMD; the incidence of type 3 neovascularization (RAP) is higher^16^, whereas the incidence of PCV/AT1 is lower than that of typical nAMD^17^. Since the choroidal perfusion status is a causative factor for MA development^26^, and low choroidal thickness is a risk factor for GA development after anti-VEGF treatment^16^, it could be speculated that the relatively high choroidal thickness of PNV might be associated with the lower incidence of MA development.

Another reason for the low incidence of MA development in PNV could be the low number of anti-VEGF injections in our study. However, in our study, there was no significant difference in terms of MA development between PNV and PCV/AT1, despite the difference in injection number. Indeed, there is still a controversy as to whether frequent anti-VEGF injections could affect MA development. Previously, the CATT study showed that monthly anti-VEGF injection had a higher risk of MA development than as-needed injection^27^. On the contrary, Recent investigations reported that MA development was not associated with the number of anti-VEGF injections between PRN and TNE regimens^28^. The effect of number of injections administered on the development of RPE atrophy should be investigated in the future.

Although the current study is the first to investigate the long-term therapeutic efficacy of anti-VEGF treatment for PNV during more than 36 months of follow-up, the study has several limitations, including its retrospective nature. First, the diagnostic criteria for PNV may not be ideal because no definite diagnostic criteria have been established so far. We defined PNV according to the most recent studies; nevertheless, a standard definition of PNV should be investigated in the future. Second, aflibercept and ranibizumab were not strictly differentiated. However, the two anti-VEGF agents have comparable visual outcomes for the treatment of neovascular AMD^29^. A recent study reported that ranibizumab and aflibercept had comparable visual outcomes for the treatment of PNV^12^. Third, we excluded the patients who underwent PDT because adjuvant PDT during anti-VEGF treatment might affect the number of injections^30^. Despite PDT, also reported as one of effective treatment modalities for PNV^31^, the long-term effect of PDT on PNV could be investigated in a future study.

In conclusion, there was no significant difference between PNV, PCV/AT1, and typical nAMD regarding visual acuity improvement after anti-VEGF treatment during 36 months. However, the injection number for PNV was significantly lower compared to PCV/AT1 and typical nAMD, and incidence of MA development was significantly lower than for typical nAMD. Further investigations regarding the differences in the pathophysiology between PNV and other types of nAMD are warranted in the future.
