# Author Correction: Long-term outcome of intravitreal anti-vascular endothelial growth factor treatment for pachychoroid neovasculopathy

**DOI:** 10.1038/s41598-021-98358-1

**Published:** 2021-09-14

**Authors:** Jihyun Yoon, Wontae Yoon, Seung kwan Na, Jihyun Lee, Chul Gu Kim, Jong Woo Kim, Han Joo Cho

**Affiliations:** Kim’s Eye Hospital, Konyang University College of Medicine, 156, 4ga, Yeoungdeungpo-dong, Yeoungdeungpo-gu, Seoul, South Korea

Correction to: *Scientific Reports* 10.1038/s41598-021-91589-2, published online 08 June 2021

The original version of this Article contained errors in the horizontal axis label of Figure 3. As a result,

"6 months, 9 months, and 12 months"

now reads:

"12 months, 24 months, and 36 months".

The original Figure 3 and accompanying legend appear below.

**Figure 3:**
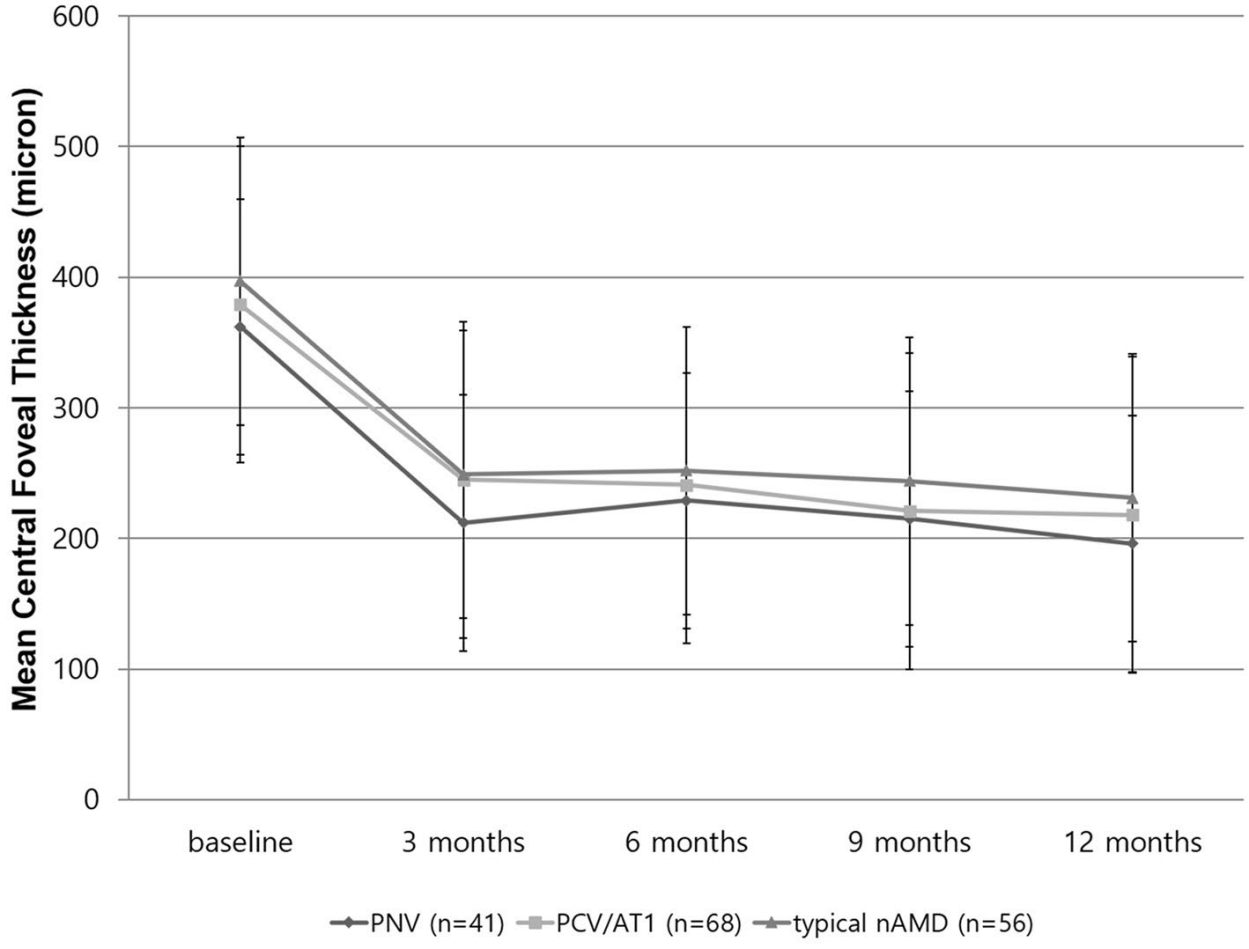
Changes in mean central foveal thickness during the 36-month anti-vascular endothelial growth factor treatment for pachychoroid neovasculopathy (PNV), polypoidal choroidal vasculopathy/aneurysmal type 1 neovascularization (PCV/AT1), and typical neovascular age-related macular degeneration (nAMD). The mean central foveal thickness showed a significant decrease when comparing from baseline through the 36 months follow-up for the groups. However, there was no significant difference between the groups in central foveal thickness at 3, 12, 24, and 36 months.

The original Article has been corrected.

